# Quality and Authenticity Evaluation of Red Yeast Rice Food Supplements from the Bulgarian Market

**DOI:** 10.3390/foods15203583

**Published:** 2026-10-09

**Authors:** Velislava Todorova, Zoya Dzhakova, Kalin Ivanov, Daniela Grekova-Kafalova, Katerina Slavcheva, Stanislava Ivanova

**Affiliations:** 1 Department of Pharmacognosy and Pharmaceutical Chemistry, Faculty of Pharmacy, Medical University of Plovdiv, 4002 Plovdiv, Bulgaria; 2 Research Institute, Medical University of Plovdiv, 4002 Plovdiv, Bulgaria; 3 Department of Organisation and Economics of Pharmacy, Faculty of Pharmacy, Medical University of Plovdiv, 4002 Plovdiv, Bulgaria

**Keywords:** monacolin K, red yeast rice, food supplements, HPLC, GC-MS/MS

## Abstract

Red yeast rice (RYR) food supplements show substantial variability in monacolin composition, raising concerns regarding product quality, labelling accuracy, and regulatory compliance. This study evaluated 14 RYR-containing food supplements purchased from the Bulgarian market using complementary HPLC-PDA, LC-MS, and GC-MS/MS approaches. A validated HPLC-PDA method was applied for quantitative determination of the lactone form of monacolin K (MK) and monascorubrin, while LC-MS was used for identity confirmation and GC-MS/MS for targeted screening of undeclared simvastatin and rosuvastatin. The measured lactone MK intake at the maximum manufacturer-recommended daily dose ranged from 0.06 to 14.11 mg/day, demonstrating substantial product-to-product variability and discrepancies between measured MK concentrations and label declarations. Three products contained lactone MK at levels of ≥3 mg/day. However, because the hydroxy acid form and other monacolins were not quantified, these results cannot be directly interpreted as total RYR-derived monacolin intake for regulatory assessment. Neither simvastatin nor rosuvastatin was detected under the applied analytical conditions. Monascorubrin was not detected in any of the analysed products under the applied analytical conditions; consequently, its non-detection cannot substantiate product authenticity, and the present findings do not support its use as a standalone authenticity marker for finished RYR supplements. Overall, the results demonstrate substantial variability in measured MK-lactone content among RYR food supplements purchased from the Bulgarian market and highlight the importance of improved analytical characterisation and product standardisation.

## 1. Introduction

Hypercholesterolemia, particularly increased levels of low-density lipoprotein (LDL) cholesterol, remains a major risk factor for the development of atherosclerotic cardiovascular disease [1,2]. Individuals with hypercholesterolemia face an approximately doubled risk of developing cardiovascular disease compared to those with normal total cholesterol levels [3]. Elevated LDL concentrations are typically managed through lifestyle modification and pharmacotherapy, most notably with statins, a widely prescribed class of lipid-lowering agents [4]. Alongside conventional treatments, various food supplements (FSs) are marketed for their purported cholesterol-lowering effects. Among the most prominent are products containing red yeast rice (RYR), which have been reported to reduce both total cholesterol and LDL-C [4,5]. Red yeast rice (RYR) is a traditional fermented product obtained by fermenting rice (*Oryza sativa* L., Poaceae) with filamentous fungi of the genus *Monascus*, most commonly *Monascus purpureus* Went. Despite the conventional term ‘red yeast rice’, *M. purpureus* is taxonomically classified as a filamentous fungus (mould), rather than a yeast [6]. During fermentation, a complex mixture of secondary metabolites is produced, including pigments responsible for the characteristic red colour of RYR, sterols, organic acids, polysaccharides, and other bioactive compounds [7]. The process also produces monacolins, including the monacolin K (MK), which possess cholesterol-lowering activity and are regarded as the key active constituents of RYR [6,8].

Monacolin K occurs in two forms: a lactone form that is structurally identical to the statin drug lovastatin, and a hydroxy acid form [9]. Products containing MK are regulated as medicinal products in the United States, whereas in the European Union, RYR preparations are marketed in food supplements, and monacolins from RYR are subject to the conditions and labelling requirements established under Annex III to Regulation (EC) No. 1925/2006, as amended by Commission Regulation (EU) 2022/860 [10]. The European Food Safety Authority (EFSA) concluded that exposure to monacolins from RYR may lead to an intake of monacolin K within the therapeutic dose range of lovastatin and that the adverse-effect profile of RYR-derived monacolins is similar to that of lovastatin. Following these safety concerns, Commission Regulation (EU) 2022/860 of 1 June 2022 amended Annex III to Regulation (EC) No. 1925/2006 by including monacolins from red yeast rice in Part B (‘Restricted substances’) and Part C (‘Substances under Union scrutiny’). Under the conditions of use established in Part B, an individual portion of a product for daily consumption must provide less than 3 mg of monacolins from RYR. Importantly, this regulatory condition applies to monacolins from RYR collectively and not specifically to the lactone form of monacolin K.

In addition to these safety considerations, RYR-containing FSs raise significant quality and safety concerns. The content of MK and other monacolins may vary markedly between products due to differences in *Monascus* strain, substrate, fermentation conditions, processing, storage, and formulation. Market surveillance studies have repeatedly shown substantial variability in monacolin content, frequent discrepancies between declared and measured amounts, and cases where monacolin content is not declared at all. Such variability may result in substantially different consumer exposure to MK among commercially available products [7].

Reported adverse effects of RYR supplements mainly affect the musculoskeletal system (myopathy, myalgia, rarely rhabdomyolysis), the liver (elevated transaminases, rarely acute hepatitis), and the gastrointestinal tract, mirroring the lovastatin profile [11,12]. Although serious events appear very rare compared with prescription statins, individual cases, including some requiring hospitalisation, have been reported at monacolin intakes as low as 3 mg/day [13]. Safety assessment is complicated by self-medication and by the large product-to-product variation in monacolin content [11,12].

Product quality assessment is further complicated by the possible occurrence of fermentation-related contaminants and undeclared pharmacologically active substances. Citrinin, a nephrotoxic mycotoxin that may be produced during Monascus fermentation, represents an additional safety concern in RYR products. However, citrinin and other fungal metabolites were not investigated in the present study and therefore fall outside the scope of the current analytical evaluation [5,14]. However, recent evidence indicates that the absence of citrinin alone is not sufficient to confirm product safety, as other toxic fungal metabolites may also occur. The analytical distinction between naturally produced MK and added synthetic lovastatin is especially challenging because the two compounds are chemically identical. Comprehensive characterisation of RYR supplements may require broader profiling of monacolins, fermentation-related metabolites, mycotoxins, and undeclared pharmacologically active substances [7]. The present study focused specifically on quantitative determination of MK-lactone, evaluation of monascorubrin, and targeted screening for selected undeclared statins.

Although previous studies have documented substantial variability in monacolin content among commercial RYR supplements, the aim of the present study was to evaluate RYR-containing food supplements purchased from the Bulgarian market by quantifying the lactone form of monacolin K (MK-lactone), comparing the measured values with analytically comparable label declarations where applicable, and screening for selected undeclared pharmacologically active substances. Because MK hydroxy acid and other monacolins were not quantified, direct assessment of compliance with the EU regulatory condition for total monacolins from RYR was outside the scope of the present analytical approach. This complementary analytical approach provides product-specific data on MK-lactone variability while explicitly considering the limitations of MK-lactone as a surrogate for total monacolin content and of conventional analytical methods for authenticity assessment.

## 2. Materials and Methods

### 2.1. Chemicals

Reference standards of monacolin K (MK) and monascorubrin (purity ≥95%) were obtained from PhytoLab GmbH & Co. KG (Vestenbergsgreuth, Germany), while simvastatin and rosuvastatin calcium (purity ≥98%) were obtained from Sigma-Aldrich (Darmstadt, Germany). LC-MS-grade acetonitrile and formic acid were obtained from Honeywell Fluka and Carl Roth, respectively.

The following hydrocarbons were used to determine the retention indices (RIs): nonane (99%), decane (≥99%), undecane (≥99%), dodecane (99%), tridecane (≥99%), tetradecane (≥99%), hexadecane (≥99%), and heptadecane (99%). These hydrocarbons were purchased from Merck KGaA, Darmstadt, Germany.

### 2.2. Samples

A total of 14 commercially available food supplements labelled as containing RYR were purchased from Bulgarian pharmacies and online retailers. Products were selected to include RYR-containing supplements available through commonly used retail channels at the time of sampling. The sampling was not intended to provide a statistically representative survey of the entire Bulgarian market; rather, it was designed as an analytical evaluation of a selection of commercially available products. The samples were stored at ambient temperature (15–25 °C), protected from light exposure, and analysed prior to their specified expiration date.

### 2.3. HPLC-PDA and LC-MS Analysis

#### 2.3.1. Preparing of Standard and Sample Solutions

Stock solutions of MK, monascorubrin, simvastatin, and rosuvastatin reference standards were prepared in acetonitrile at a concentration of 1 mg/mL. An ultrasonic bath (Bandelin, Berlin, Germany) was used to facilitate dissolution, and the solutions were stored in amber vials at 4 °C protected from light until analysis. Working standard solutions were prepared by dilution with acetonitrile. The calibration ranges were 3.125–50 μg/mL for MK and 5–75 μg/mL for monascorubrin.

The analytical procedure specifically quantified MK-lactone using an authentic lactone reference standard. MK hydroxy acid, other monacolins, including dehydromonacolin K, and total monacolins were not quantified, and no conversion between the lactone and hydroxy acid forms was performed. Consequently, the measured MK-lactone represented only one component of the total monacolin content and was not used independently to assess compliance with the EU regulatory condition for total monacolins from RYR.

For each commercial product, three individual dosage units (tablets or capsules) were independently prepared and analysed (n = 3). Tablets were crushed and homogenised, while capsule contents were homogenised before extraction. Each dosage unit was extracted with 5 mL of acetonitrile and sonicated for 30 min at room temperature. The extracts were filtered through 0.22 μm PTFE membrane filters prior to analysis.

The filtered extracts were diluted with acetonitrile as necessary to ensure that the analytical response fell within the validated calibration range. Samples exceeding the upper calibration limit were further diluted and reanalysed, with the corresponding dilution factors included in the final calculations. All reported MK-lactone concentrations were obtained from measurements within the validated range of 3.125–50 μg/mL.

MK-lactone content was calculated per dosage unit and expressed as the amount corresponding to the maximum manufacturer-recommended daily dose. Quantitative results were calculated from three independently prepared dosage units and are reported as mean ± standard deviation (SD) and relative standard deviation (RSD, %).

#### 2.3.2. Instrumentation

An LC40-PDA instrument (Shimadzu, Kyoto, Japan) was employed for method development and HPLC-PDA analysis, while LC-MS analysis utilized an LC-MS 2050 system from the same manufacturer. Separation was achieved using a Shim-pack C18 column (150 mm × 4.6 mm, 3 μm) (Schimadzu, Kyoto, Japan).

#### 2.3.3. Method Development

The initial stage of method development focused on selecting the optimal mobile phase, consisting of water and acetonitrile. Various ratios of these solvents were systematically evaluated to identify the most suitable composition. An isocratic mixture of 30% water and 70% acetonitrile, delivered at a flow rate of 1.0 mL/min was found to provide well-shaped and symmetrical peaks. The analysis was completed within 15 min. The column was maintained at 40 °C, and the injection volume was set to 10 µL. UV–VIS spectra were acquired over the range of 200–800 nm. MK was evaluated at its characteristic absorption maximum of approximately 239 nm, whereas monascorubrin was specifically evaluated at 473 nm, corresponding to its experimentally observed absorption maximum. The recorded spectra are presented in Figure S1.

The corresponding retention times were 5.179 min for MK and 9.920 min for monascorubrin. Data collection and processing were conducted using LabSolutions software (version 5.118, Shimadzu, Kyoto, Japan).

For LC-MS characterization, the same column, column temperature, organic-phase proportion, flow rate, and isocratic elution mode as in the HPLC-PDA method were used. However, the aqueous component of the LC-MS mobile phase contained 0.1% formic acid to improve ionization efficiency. The mass spectrometer was equipped with a single quadrupole detector operating with a DUIS interface at 3 kV. Gas flow rates were set at 2 L/min for nebulizing gas, 5 L/min for drying gas, and 7 L/min for heating gas, while the desolvation temperature was maintained at 450 °C. Full-scan (TIC) data were recorded over an *m*/*z* range of 100–650. Selected ion monitoring (SIM) was performed for the following ions: rosuvastatin (*m*/*z* 482, 504), MK (*m*/*z* 405, 422, 427), simvastatin (*m*/*z* 419, 441), and monascorubrin (*m*/*z* 383, 401) with their respective retention times: 1.86 min, 5.232 min, 6.622 min and 10.029 min, respectively. Data acquisition and analysis were carried out using LabSolutions software v 5.118 (Shimadzu, Kyoto, Japan).

The LC-MS analysis of simvastatin and rosuvastatin was performed as a targeted qualitative screening procedure. Analyte-specific LOD and LOQ values and matrix-spike recoveries were not established for these compounds. Consequently, non-detection was interpreted as the absence of a detectable signal under the applied analytical conditions rather than as definitive evidence of analyte absence.

#### 2.3.4. Validation of HPLC-PDA Method

The developed HPLC-PDA method was validated according to the International Council for Harmonisation of Technical Requirements for the Registration of Medicinal Products for Human Use with the following validation parameters: linearity, range, precision, accuracy, limits of detection (LOD), limits of quantification (LOQ), and robustness [15].

##### Linearity

The quantification of MK and monascorubrin was performed utilizing an external standard calibration approach. For MK, a standard solution was prepared at five distinct concentration levels spanning from 3.125 to 50 μg/mL, with each concentration injected in triplicate to establish the calibration curves. Similarly, monascorubrin was prepared across a range of five concentrations from 5 to 75 μg/mL. The linearity of the developed method was rigorously evaluated by correlating the integrated peak areas with the known concentrations of the respective standard solutions. To statistically assess this linearity, the coefficient of determination (R^2^) was calculated.

##### Accuracy and Precision

The accuracy of the proposed analytical method was evaluated by determining the percentage recovery, expressed as the difference between the experimentally obtained mean concentration and the corresponding theoretical value. Three quality control levels were employed for each analyte, namely low (5 μg/mL), medium (10 μg/mL), and high (25 μg/mL). All measurements were conducted in quintuplicate at each concentration level to ensure statistical robustness.

The precision of the method was assessed in terms of intra-day (repeatability) and inter-day (intermediate precision) variability. Intra-day precision was evaluated by analysing freshly prepared samples at the three quality control levels, each in five replicates within a single day. Inter-day precision was determined by analyzing freshly prepared samples at the same concentration levels over three consecutive days, with five replicates per level.

##### Robustness

Method robustness was assessed by evaluating the impact of deliberate variations in column temperature on chromatographic performance. The temperature was systematically adjusted within a range of 38 to 42 °C, during which the analyte retention times were recorded.

##### Stability

The stability of the standard solutions was evaluated following storage at 2–8 °C for a duration of one week. The solutions were initially inspected visually to ensure clarity and the absence of precipitation, and were subsequently analyzed using the developed method. Stability was determined by comparing the chromatograms of freshly prepared solutions with those of the stored samples and by calculating the percentage recovery.

#### 2.3.5. Matrix Effect Assessment of HPLC-PDA Method

Matrix effects were assessed using a post-extraction spiking approach. Extracted samples were spiked with known concentrations of analytes at three levels (10, 20, and 30 μg/mL). For MK, a sample containing endogenous levels of the analyte (Sample 5) was used. To account for the native content, the matrix effect was calculated by subtracting the peak area of the non-spiked sample from that of the spiked sample. For monascorubrin, a representative sample without detectable endogenous interference (Sample 4) was used for evaluation. Matrix effects were calculated by comparing the peak areas obtained from spiked matrix extracts with those from standard solutions prepared in solvent at equivalent concentrations.

Dedicated positive and negative matrix quality-control samples were not systematically included throughout the analytical sequence, which represents a limitation of the targeted qualitative screening.

#### 2.3.6. Statistical Analysis

Statistical calculations were performed using Microsoft Excel 2024 (Microsoft Corporation, Redmond, WA, USA). Quantitative results for the commercial food supplements are expressed as mean ± standard deviation (SD) from three independently prepared and analysed dosage units (n = 3). The coefficient of variation (CV, %) was calculated as (SD/mean) × 100 and was used to describe the variability among replicate measurements.

Method-validation data were evaluated using the number of replicate measurements specified for each validation parameter. Linearity was assessed by linear regression of analyte peak area against concentration, and the corresponding regression equations and coefficients of determination (R^2^) were calculated. Accuracy was evaluated as percentage recovery at three concentration levels (5, 10, and 25 μg/mL), with five replicate measurements at each level (n = 5). Precision was evaluated as intra-day repeatability and inter-day intermediate precision and is reported as mean ± SD and CV (%). Intra-day precision was determined from five replicate measurements at each concentration level within one day, whereas inter-day precision was evaluated over three consecutive days using five replicate measurements per concentration level per day. The quantitative results for the commercial products were evaluated descriptively, and no inferential statistical comparisons between products were performed.

### 2.4. GC-MS/MS

#### Chromatographic Conditions

The gas chromatography–mass spectrometry (GC-MS/MS) analysis was performed on a Bruker Scion 436-GC SQ MS system (Bremen, Germany) fitted with a capillary Zebron ZB-5MSplus column (30 m × 0.25 mm, 0.25 μm film thickness, Torrance, CA, USA). The used software was v. 8.2. The oven temperature used was according to the previously reported protocol by Todorova et al. [16]. The oven temperature was initially kept at 90 °C for 1 min and then increased to 190 °C at 15 °C/min and after that increased to 300 °C at 5 °C/min and then kept for 1 min. Helium was the carrier gas with a flow rate of 1 mL/min. The mass spectra were collected over the range of *m*/*z* 50–450 in full scan mode and the ionization energy for the mass was 70 eV. The temperatures of the detector and injector were 300 and 250 °C, respectively. The injection volume was 1 μL and a split ratio 1:20 was used. The MS spectra of lovastatin was compared with its spectral data from Wiley NIST11 Mass Spectral Library [17]. Subsequently, a targeted multiple reaction monitoring (MRM) analysis was performed for simvastatin, MK, and rosuvastatin in order to enhance selectivity and confirm their presence or absence in the analysed samples. The optimized MRM transitions for simvastatin, MK and rosuvastatin are presented in Table 1.

The GC-MS/MS analysis was used as a targeted qualitative screening approach rather than as a validated quantitative method for rosuvastatin determination. No derivatization procedure was applied, and analyte-specific validation parameters such as recovery, accuracy, precision, LOD, and LOQ were not established for rosuvastatin under the applied GC-MS/MS conditions. Therefore, the absence of a detectable rosuvastatin signal was interpreted only within the limitations of the applied screening conditions and was not considered proof of its absolute absence. Rosuvastatin was additionally included in the targeted LC-MS screening to provide complementary analytical evidence.

## 3. Results and Discussion

### 3.1. HPLC-PDA Method Performance and Validation

#### 3.1.1. Method Performance

A rapid and sensitive HPLC-PDA method was developed for the precise quantification of MK and monascorubrin in FSs. Using this optimized method, MK and monascorubrin were clearly resolved, with retention times of 5.179 min and 9.92 min, respectively (Figure 1). These retention times demonstrated excellent reproducibility, with relative standard deviations not exceeding 0.2%.

A system suitability test was performed to evaluate the operational performance of the analytical system and the effectiveness of the chromatographic separation. This assessment involved the determination of several critical parameters: selectivity factor (α), resolution (Rs), number of theoretical plates (N), and symmetry factor (As) (Table S1). These indices were calculated in strict accordance with the guidelines established by the United States Pharmacopeia [18]. The data obtained confirmed that the chromatographic system satisfied the necessary requirements for selectivity and efficiency, as evidenced by the distinct discrimination of the peaks. Owing to its accessibility, rapid execution, and procedural simplicity, the proposed method was subsequently subjected to validation.

#### 3.1.2. Method Validation

##### Linearity

As summarized in Table 2, the results demonstrated a high degree of linearity.

##### Accuracy and Precision

The obtained recovery values demonstrated a high degree of accuracy of the method across the investigated concentration range. A summary of the accuracy results for the analysed compounds is presented in Table 3.

The results from precision indicated low variability, with relative standard deviation values within acceptable limits, confirming the high precision of the developed method. A detailed summary of the precision data is provided in Table 4.

The correlation coefficients were found to be close to unity, and together with the high recovery percentages and low standard deviation values, confirm that the method exhibits excellent linearity, accuracy, and precision. These findings collectively demonstrate the reliability and suitability of the proposed analytical procedure for the quantitative determination of MK and monascorubrin.

##### Limit of Detection and Quantification

The limit of detection and limit of quantification were derived from the linearity data, utilizing the standard deviation of the linear response (σ) in relation to the slope of the calibration curve (S) with the following equitation’s: LOD = 3.3*σ/S and LOQ = 10*σ/S. These two threshold limits were established according to the statistical parameters presented in Table 2.

##### Robustness

No significant alterations in resolution were observed, suggesting that minor temperature fluctuations did not adversely affect the separation efficiency, thereby confirming the inherent robustness of the analytical method. Moreover, the robustness of the method was further evaluated by introducing small deliberate variations in the mobile phase composition (±2%) and flow rate. These modifications did not result in any significant changes in analyte retention times, indicating that the method remains stable and reliable under slight variations in chromatographic conditions.

##### Stability

The results indicated that the standards remained stable throughout the storage period, with no evidence of degradation or shifts in chromatographic behavior. Autosampler stability was evaluated by reanalyzing processed samples after 24 h storage in the autosampler at 15 °C. The measured concentrations were compared with those obtained from freshly prepared samples. These findings support the feasibility of reliable storage and subsequent analysis without a loss of chemical integrity.

##### Matrix Effect

For MK, the matrix effect values were 100.09%, 104.55%, and 105.48% at 10, 20, and 30 μg/mL, respectively, indicating a slight signal enhancement across the studied concentration range. In the case of monascorubrin, matrix effect values of 100.45%, 91.76%, and 95.42% were obtained, corresponding to negligible to moderate signal suppression. Although matrix effects were evaluated using a single representative sample, the investigated food supplements differed in excipient composition and formulation, which may influence analyte response to a certain extent. Nevertheless, the observed matrix effects remained within acceptable limits, suggesting that such variability did not significantly affect the performance of the developed HPLC–PDA method. Future studies including a wider range of representative matrices would provide a more comprehensive evaluation of matrix-related effects.

Peak purity analysis was performed for MK in all analysed red yeast rice FS samples (n = 14). In all cases, the purity angle values were lower than the corresponding purity thresholds, confirming spectral homogeneity of the analyte peak and absence of co-eluting interferences. These results demonstrate the specificity of the developed HPLC–PDA method in complex supplement matrices. Representative peak purity plots for Sample 5 and Sample 12 are provided in Figure S2.

### 3.2. LC-MS

The LC–MS method was further developed and applied for the detection and confirmation of rosuvastatin, simvastatin, MK, and monascorubrin in the FSs containing RYR. The method enabled reliable identification of all target analytes using both TIC and SIM modes (Figure 2 and Figure 3).

Each compound was identified based on its characteristic mass spectral ions. MK was confirmed by the presence of ions at *m*/*z* 405, 422, and 427, while monascorubrin exhibited ions at *m*/*z* 383 and 401. Rosuvastatin and simvastatin were identified by their respective ions at *m*/*z* 482 and 504, and *m*/*z* 419 and 441. The retention times of all analytes were consistent with those of the corresponding reference standards analyzed under identical chromatographic conditions, as follows: rosuvastatin (t_R_ = 1.860 min), MK (t_R_ = 5.235 min), simvastatin (t_R_ = 6.621 min) and monascorubrin (t_R_ = 10.024 min). The application of SIM mode improved method selectivity, allowing for clear discrimination of the analytes from potential matrix components. No significant interfering signals were observed at the retention times of the investigated compounds, indicating good specificity of the method.

### 3.3. Quantification of Monacolin K and Monascorubrin in Commercial RYR

The quantified MK content exhibited marked variability across the analysed RYR FSs as shown in Table 5 and Figure S3, ranging from as little as 0.06 mg to as much as 14.11 mg per maximum recommended daily dose.

Notably, three products contained lactone MK at measured levels of ≥3 mg/day (Samples 10, 12, and 14). However, the regulatory restriction applies to total monacolins from RYR rather than specifically to the lactone form of MK. Because MK hydroxy acid and other monacolins were not quantified, direct assessment of compliance with the total-monacolin regulatory limit was not possible. The highest measured MK-lactone content was observed in Sample 12 (14.11 mg/day), although no quantitative MK amount was declared on its label. These findings demonstrate substantial variability in MK-lactone content and differences between measured values and product label information. However, the present analytical study was not designed to demonstrate actual adverse health effects or quantify consumer safety risk. Moreover, because MK hydroxy acid, other monacolins, citrinin, and other potentially relevant constituents were not determined, the results should not be interpreted as a comprehensive safety assessment.

Substantial discrepancies were identified between declared and analytically determined MK levels. Several products, including Samples 1, 4, 6, 9, and 11, contained considerably lower amounts than those indicated by the manufacturer. Conversely, Sample 14 exhibited more than double the declared MK content. In a number of cases, only red yeast rice or total monacolin content was reported, with no specific reference to MK, thereby limiting transparency and hindering accurate safety evaluation. Furthermore, a subset of samples (notably Samples 2, 3, 7, 8, 12, and 13) failed to provide explicit information regarding MK content, despite containing RYR or monacolins. Such omissions may result in inconsistent dosing and unpredictable consumer exposure. Additionally, the very low MK levels observed in certain products (e.g., Samples 5 and 13) raise concerns regarding their therapeutic efficacy and the degree of product standardisation.

Collectively, these findings underscore pronounced heterogeneity in the quality and standardisation of RYR-containing food supplements. The observed variability and differences between measured and declared MK-lactone values may reflect multiple factors, including raw-material composition, fermentation and manufacturing conditions, processing, storage-related degradation, and differences in product age. Analytical variability may also contribute to the observed differences; however, the present study was not designed to determine the relative contribution of these individual factors. All products were analysed before their stated expiration dates and were stored at 15–25 °C protected from light after purchase. However, manufacturing dates and pre-purchase storage histories were not systematically available; therefore, the potential influence of product age and storage before purchase could not be assessed. Moreover, given that MK is chemically identical to lovastatin, products exceeding the 3 mg/day regulatory limit may expose consumers to pharmacologically significant statin-like effects, particularly in the absence of medical supervision. The inconsistencies observed between labelled and measured MK contents highlight the urgent need for more rigorous quality control measures, enhanced labelling accuracy, and routine analytical verification to ensure consumer safety and compliance with regulatory standards.

An additional analytical verification step was performed using LC–MS in the tested samples. MK was unequivocally identified, confirming the reliability and specificity of the applied method. In contrast, monascorubrin was not detected in any of the analysed samples. This finding may be explained by several factors. Monascorubrin is chemically unstable and may degrade during processing, drying, extraction, encapsulation, storage, or prolonged exposure to oxygen and light. In addition, its concentration is strongly influenced by the *Monascus* strain, fermentation conditions, substrate composition, and downstream manufacturing processes. Commercial production may also involve partially purified red yeast rice extracts that retain monacolin K while containing only trace amounts of fungal pigments. Furthermore, ionisation efficiency under the applied LC–MS conditions may have further reduced its detectability. Therefore, the absence of monascorubrin does not necessarily indicate adulteration but rather reflects the variability in commercially available RYR products. These findings suggest that monascorubrin is not a sufficiently robust standalone authenticity marker for finished food supplements and should be complemented by additional chemical markers or advanced analytical approaches, such as stable isotope analysis. Importantly, the present study does not validate monascorubrin as an authenticity marker. Validation of such a marker would require demonstration of its consistent occurrence in authentic RYR materials and finished products, evaluation of its stability during processing and storage, and assessment of its specificity relative to non-authentic or adulterated products. Because monascorubrin was not detected in any of the products investigated here, its absence cannot be used as evidence either for or against authenticity. The present findings therefore indicate that monascorubrin alone has limited value for authenticity assessment of finished RYR food supplements.

For samples exhibiting the highest MK concentrations (Samples 12 and 14), further advanced analytical investigations—such as stable isotope ratio analysis, chiral chromatography, or nuclear magnetic resonance spectroscopy—are warranted to elucidate the origin of MK and to determine whether it is derived from natural fermentation processes or potentially from synthetic or semi-synthetic sources. Additionally, targeted screening for synthetic statins revealed no detectable levels of simvastatin or rosuvastatin in any of the tested samples. These results further support the specificity of MK as the principal statin-like compound present and exclude the occurrence of commonly used synthetic statins within the detection limits of the method.

### 3.4. GC-MS/MS Screening for Undeclared Synthetic Statins

To further ensure the identity of the target compounds and to screen for potential adulterants or secondary metabolites, a GC-MS/MS analysis was conducted. The volatile profiles of the extracts were characterized using full-scan mode, while high sensitivity and selectivity for the target statins were achieved through MRM. The analysis revealed the presence of MK in all analysed samples, as confirmed both by TIC screening (Figure S4) and by targeted MRM transitions. In contrast, the investigated synthetic statins, namely rosuvastatin and simvastatin, were not detected in any of the samples. These results indicate that MK was the only statin-like compound identified under the applied experimental conditions. This finding supports the authenticity of the analysed products with respect to their natural origin and suggests the absence of adulteration with commonly used synthetic statins, although it does not preclude the presence of other structurally related or unknown compounds not included in the targeted analysis.

### 3.5. Comparison with Previous Studies

A recent evaluation of RYR FSs on the European market found that several products contained more than 3 mg of monacolins, raising potential safety concerns. While no synthetic statins were detected and all samples complied with citrinin limits, some products were suspected of adulteration with lovastatin [19].

The results of the present study are consistent with a substantial body of literature demonstrating marked variability in the composition, quality, and safety of RYR supplements. Previous investigations have reported wide ranges in MK content across commercially available products, reflecting a lack of standardisation in manufacturing practices. Reported MK levels vary from 0.09 mg to 5.48 mg per 1200 mg of RYR, with many products failing to declare MK content [20]. Other studies have found concentrations ranging from 1.51 to 9.88 mg per serving [21] and up to 10.09 mg per capsule [22], in some cases exceeding recommended safety limits. For comparison, the present study included 14 RYR food supplements and found MK-lactone contents ranging from 0.06 to 14.11 mg at the maximum manufacturer-recommended daily dose. Direct numerical comparison should, however, be interpreted cautiously because the present study quantified MK-lactone only, whereas the analytes and reporting units differed among previous investigations. In agreement with the present findings, discrepancies between labelled and measured MK content appear to be widespread. Reported deviations range from 1.9% to 153.5% of declared values [23], with some products containing substantially higher or lower amounts than indicated, or none at all. Similar inconsistencies have been observed using advanced analytical techniques such as UHPLC–MS and ^1^H-NMR, where only a minority of products complied with their label claims [24]. Beyond MK, several studies have highlighted the complex and variable composition of monacolins and related compounds in RYR supplements. Minor monacolins and degradation products can constitute up to 37% of the total monacolin content [25], and their relative distribution may vary depending on overall MK levels, with certain derivatives predominating in high-MK products [21]. This compositional variability complicates both quality assessment and safety evaluation.

The present study quantified only the lactone form of monacolin K. Since the hydroxy acid form was not determined, the lactone-to-hydroxy acid ratio could not be evaluated. This ratio has been suggested as an additional marker for differentiating naturally fermented RYR products from products potentially adulterated with synthetic lovastatin and should therefore be included in future investigations. Simvastatin and rosuvastatin were selected as targets for two reasons. First, simvastatin is a semi-synthetic derivative of lovastatin, differing by one additional methyl group on the side chain; so, it is structurally close to MK and is a plausible undeclared pharmaceutical statin in MK-standardised products; rosuvastatin was included as a widely prescribed, fully synthetic statin. Second, the only study we identified that reported simvastatin in RYR supplements found it at trace levels (0.1–7.5 µg per daily dose) in 30 of 37 products [14]; the authors did not establish whether this reflects deliberate adulteration, and an independent screening of 35 RYR supplements [19] did not detect synthetic statins. The simvastatin evidence therefore rests on a single report and should not be taken as evidence that it is a common adulterant. Our targeted screening was designed to check this question for the Bulgarian market, and its negative result applies only to the compounds and conditions tested. Moreover, adulteration and contamination of RYR supplements have been widely reported, with some studies identifying the presence of synthetic statins, including lovastatin and trace levels of simvastatin, raising concerns about intentional adulteration [14,26]. In contrast, in the present study, no detectable signals corresponding to simvastatin or rosuvastatin were observed under the applied targeted screening conditions. However, this finding should not be interpreted as evidence of product authenticity or natural origin, because fermentation-derived MK-lactone and externally added lovastatin are chemically identical and cannot be distinguished using the analytical approaches applied in the present study. The detection of such adulteration is complicated by the structural identity of MK and commercially produced lovastatin, which cannot be reliably distinguished using routine analytical methods such as HPLC, HPLC–MS/MS, and GC–MS [9,27]. Previous reports suggest that pharmaceutical lovastatin may be illegally added to RYR products to artificially increase monacolin content for economic gain [14,27,28]. To address this challenge, [29] proposed that the proportional relationship between the lactone and acid forms of MK can serve as an indicator of adulteration, an approach later applied by [14] who suggested the presence of externally sourced MK and detected simvastatin in certain samples. More recently, stable carbon isotope (δ^13^C) analysis has emerged as a reliable tool for detecting adulteration, enabling differentiation between natural and synthetic sources following chromatographic isolation and EA–IRMS analysis. Using a δ^13^C threshold of −28.3‰, the authors identified several samples with isotopic signatures indicative of non-natural origin, demonstrating the effectiveness of this approach. Another important safety concern is the presence of the mycotoxin citrinin. Several studies have reported its frequent occurrence in RYR products, occasionally at levels exceeding regulatory limits [14,22,30], although other investigations have found no detectable contamination [26]. Although citrinin was not a primary focus of the present study, its documented variability further emphasises the need for comprehensive quality control.

Overall, the findings of the present study are in strong agreement with previous literature, confirming that RYR food supplements are characterised by significant heterogeneity in composition, labelling accuracy, and safety profile. This persistent variability underscores the need for stricter regulatory oversight, harmonised analytical methodologies, and improved manufacturing standardisation to ensure consumer protection.

From a clinical perspective, the substantial variability in MK-lactone exposure observed among the analysed products is relevant because consumers using different RYR supplements according to the manufacturers’ recommended daily doses may be exposed to markedly different amounts of pharmacologically active MK-lactone. The observed discrepancies between label information and analytically determined MK-lactone content may further complicate estimation of actual consumer exposure. Nevertheless, the present study did not assess clinical outcomes, and no conclusions regarding the efficacy or safety of individual products can be drawn from the analytical data alone.

From a regulatory perspective, three products provided ≥ 3 mg/day of measured MK-lactone at the maximum recommended daily dose. Neither compliance nor exceedance of the 3 mg/day limit can be equated with the presence or absence of a safety concern; the limit reflects the regulatory decision in Regulation (EU) 2022/860, whereas the dose–response relationship for monacolin adverse effects remains poorly characterised. However, Regulation (EU) 2022/860 applies to total monacolins from RYR, whereas the present analytical method quantified only the lactone form of MK. Therefore, the measured MK-lactone values cannot be used alone to establish regulatory compliance or non-compliance. The findings instead highlight the importance of analytical methods capable of characterising multiple monacolin forms, together with clear and accurate product labelling, for the quality control and regulatory assessment of RYR food supplements.

The present study has several limitations. The number of analysed food supplements was relatively limited and restricted to products available on the Bulgarian market, which may not fully reflect the variability in RYR supplements in other regions. In addition, the determination of citrinin and other fungal metabolites was beyond the scope of the present study. Therefore, the present work focused on product quality, authenticity, and regulatory compliance rather than providing a comprehensive safety assessment. The distinction between naturally occurring monacolin K and externally added lovastatin also remains analytically challenging. FMK-lactone is chemically identical to lovastatin, and the chromatographic and mass-spectrometric methods applied in this study cannot distinguish fermentation-derived MK-lactone from externally added pharmaceutical lovastatin. Consequently, detection of MK-lactone cannot by itself substantiate the natural origin or authenticity of the product, and the present analytical workflow cannot exclude adulteration with externally added lovastatin. Source-discriminating approaches, such as stable-isotope analysis, would be required to address this question.

Future studies should include a larger number of products and batches from different markets and incorporate comprehensive profiling of MK-lactone, MKA, other monacolins, Monascus-derived pigments, and other fermentation-related metabolites. Where assessment of the origin and authenticity of MK is required, source-discriminating approaches, such as stable-isotope analysis, should also be considered.

## 4. Conclusions

The present study demonstrated substantial variability in the lactone MK content of RYR food supplements available on the Bulgarian market, together with differences between analytically determined concentrations and product label information. Measured lactone MK exposure at the maximum manufacturer-recommended daily dose ranged from 0.06 to 14.11 mg/day, with three products containing ≥3 mg/day of lactone MK. Because the hydroxy acid form of MK and other monacolins were not quantified, these values represent a lower bound of total monacolin content and cannot be directly equated with total RYR-derived monacolin intake for regulatory compliance assessment. No simvastatin or rosuvastatin was detected under the applied targeted analytical conditions; however, their absence does not establish the natural origin of MK or exclude other forms of adulteration. No detectable signals corresponding to simvastatin or rosuvastatin were observed in the analysed samples under the applied targeted screening conditions. However, this finding does not establish the natural origin or authenticity of the detected MK. Fermentation-derived MK-lactone and externally added lovastatin are chemically identical and cannot be distinguished using the analytical approaches applied in the present study. Therefore, the absence of detectable simvastatin and rosuvastatin should be interpreted only as a negative result for these specifically targeted compounds under the applied analytical conditions and not as evidence of product authenticity.

## Figures and Tables

**Figure 1:**
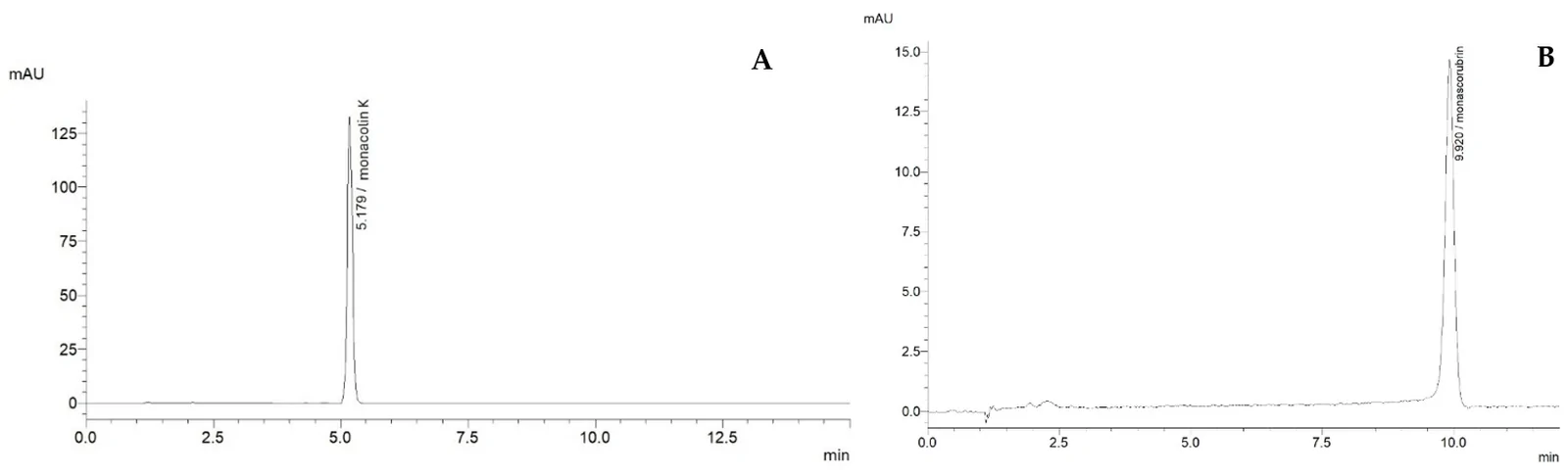
Representative HPLC-PDA chromatograms of (**A**)—MK (40 µg/mL) and (**B**)–monascorubrin (40 µg/mL).

**Figure 2:**
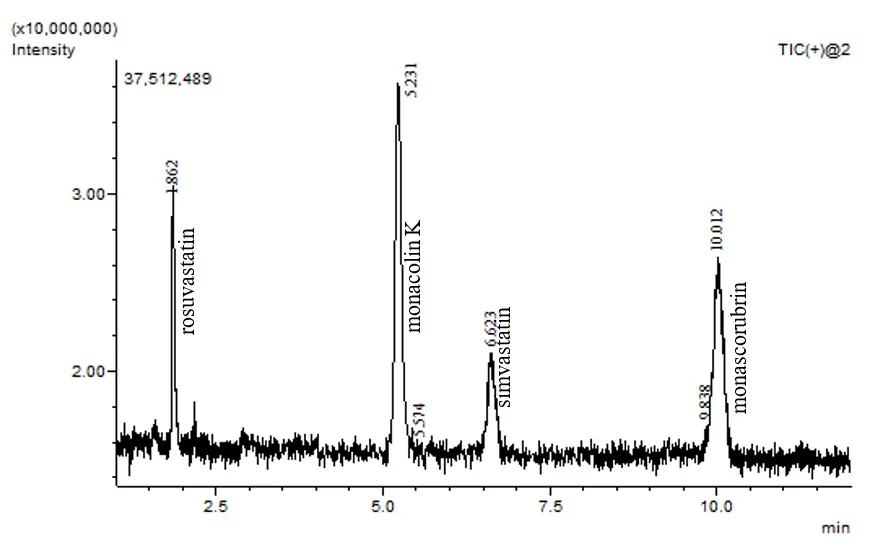
TIC chromatogram of rosuvastatin, MK, simvastatin and monascorubrin.

**Figure 3:**
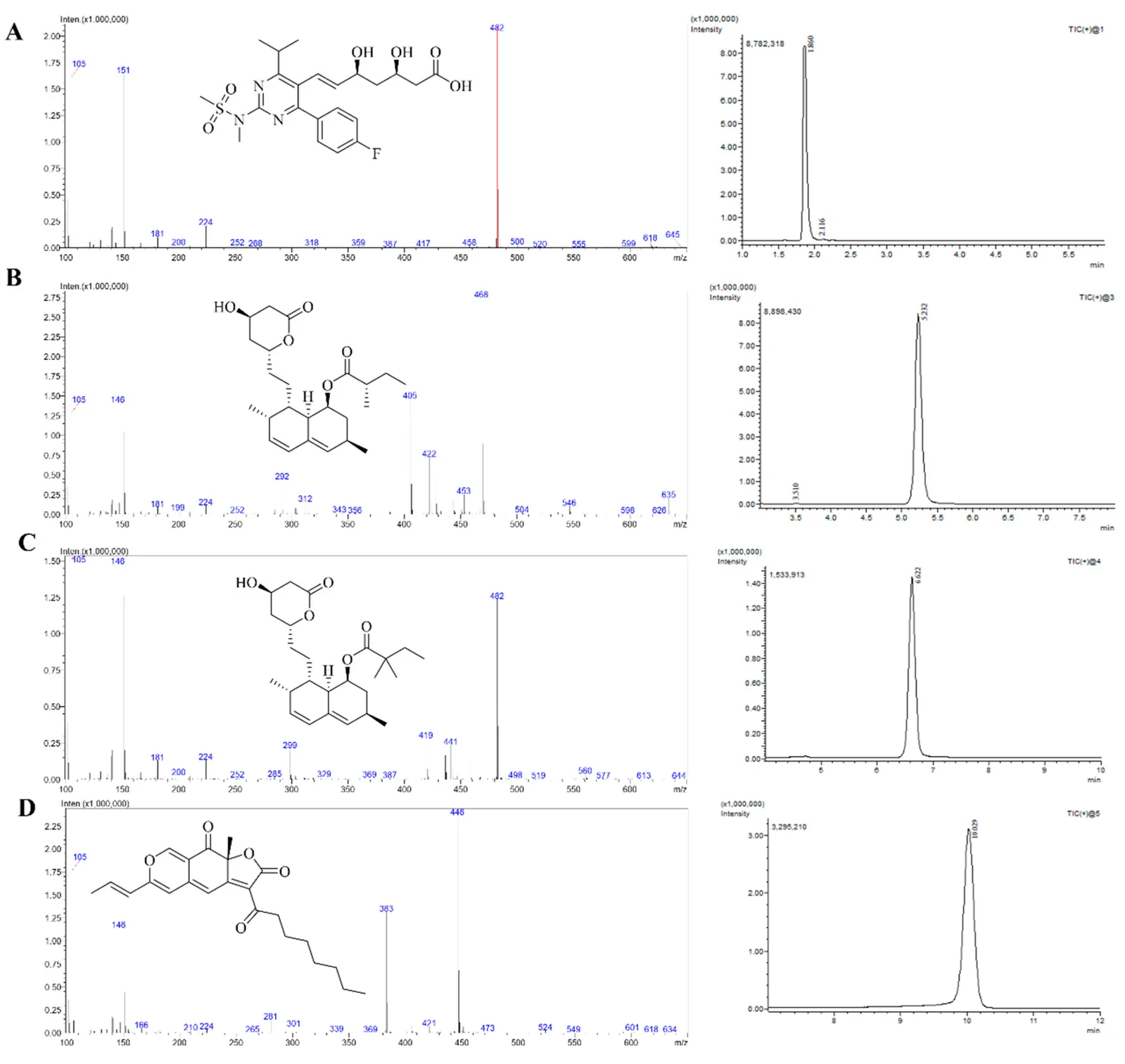
SIM chromatogram and mass spectra of (**A**) rosuvastatin, (**B**) MK, (**C**) simvastatin and (**D**) monascorubrin.

**Table 1:** Optimized MRM transitions parameters for target molecules.

Compound	Retention Time	Precursor Ion (*m*/*z*)	Product Ion (*m*/*z*)	Collision Energy (eV)	Type
Simvastatin	30.12	418	199	15	Qualifier
		199	181	15	Quantifier
		181	159	15	Qualifier
Monacolin K	26.66	404	198	15	Qualifier
		198	159	15	Quantifier
		159	57	15	Qualifier
Rosuvastatin	23.83	482	258	15	Quantifier
		288	258	15	Qualifier

**Table 2:** Evaluation of the linearity, LD, and LQ of the developed HPLC-PDA method for determination and quantification of MK and monascorubrin.

Parameter	Monacolin K	Monascorubrin
Linear regression line	y = 35,975x + 10,599	y = 23,555x – 35,538
R^2^	0.9999	0.9999
LOD	0.61 µg/mL	1.09 µg/mL
LOQ	1.84 µg/mL	3.30 µg/mL

**Table 3:** Evaluation of the accuracy of the developed HPLC-PDA method for the detection and quantification of MK and monascorubrin.

Concentration (μg/mL)	Mean (μg/mL ± SD)	Recovery%	CV%
**Monacolin K**			
25	25.17 ± 0.25	100.67	1.00
10	9.96 ± 0.02	99.60	0.21
5	4.99 ± 0.02	99.85	0.30
**Monascorubrin**			
25	24.92 ± 0.06	99.68	0.24
10	9.93 ± 0.03	99.22	0.29
5	5.01 ± 0.01	100.15	0.19

**Table 4:** Evaluation of the precision of the developed HPLC-PDA method for the detection and quantification of MK and monascorubrin.

Concentration (μg/mL)	Intra-Day Precision			Inter-Day Precision		
	Mean (μg/mL ±SD)	SEM	CV%	Mean (μg/mL ±SD)	SEM	CV%
**Monacolin K**						
25	24.95 ± 0.20	0.09	0.84	25.11 ± 0.16	0.06	0.64
10	10.02 ± 0.30	0.01	0.33	10.02 ± 0.90	0.04	0.91
5	4.99 ± 0.01	0.01	0.32	5.01 ± 0.03	0.01	0.62
**Monascorubrin**						
25	24.97 ± 0.12	0.05	0.47	24.89 ± 0.27	0.11	1.10
10	9.92 ± 0.04	0.02	0.42	9.90 ± 0.04	0.01	0.40
5	4.99 ± 0.01	0.01	0.23	5.06 ± 0.03	0.01	0.68

**Table 5:** Information provided on the label, actual content of monacolin K and synthetic statins found in the FSs with measured MK using LC-PDA.

Sample N°	Country of Origin	Maximum Daily Dose	Declared RYR (mg/day)	Declared MK (mg/day)	Measured MK Amount (mg/day)	Measured MK-Lactone ≥ 3 mg/day
Sample 1	Germany	2 Tablets	96.6 mg RYR	2.95 mg	0.77 ± 0.08 mg	No
Sample 2	USA	1 Capsule	600 mg RYR	2.4 mg	0.71 ± 0.08 mg	No
Sample 3	Spain	1 Capsule	58 mg RYR	2.9 mg	1.05 ± 0.10 mg	No
Sample 4	Switzerland	1 Capsule	72.5 mg RYR	2.9 mg	1.41 ± 0.13 mg	No
Sample 5	Bulgaria	2 Tablets	600 mg RYR	Under 3 mg	0.06 ± 0.2 mg	No
Sample 6	Italy	1 Capsule	2.85 mg Monacolins	2.15 mg	0.71 ± 0.8 mg	No
Sample 7	Italy	2 Tablets	72.5 mg RYR (2.9 mg Monacolins)	n.d. *	2.17 ± 0.18 mg	No
Sample 8	Bulgaria	1 Tablet	240 mg RYR (2.4 mg Monacolins)	n.d. *	2.73 ± 0.23 mg	No
Sample 9	Poland	1 Tablet	n.d. *	2.9 mg	0.96 ± 0.24 mg	No
Sample 10	Poland	1 Tablet	99.67 mg RYR	2.99 mg	3.01 ± 0.14 mg	Yes
Sample 11	Italy	1 Capsule	200 mg RYR	standardized to 1.5% lovastatin (3 mg)	0.90 ± 0.09 mg	No
Sample 12	Bulgaria	2 Capsules	800 mg RYR	n.d. *	14.11 ± 0.56 mg	Yes
Sample 13	USA	2 Capsules	1200 mg RYR	n.d. *	0.08 ± 0.03 mg	No
Sample 14	Italy	1 Capsule	2.9 mg Monacolins	2.2 mg	5.00 ± 0.42 mg	Yes

* n.d. = not declared. The ≥3 mg/day classification refers exclusively to the experimentally determined MK-lactone content. It should not be interpreted as a direct assessment of compliance with the EU regulatory condition, which applies to total monacolins from red yeast rice.

## Data Availability

The original contributions presented in this study are included in the article/Supplementary Materials. Further inquiries can be directed to the corresponding author.

## Supplementary Materials

The following supporting information can be downloaded at: https://www.mdpi.com/article/10.3390/foods15203583/s1, Table S1. System suitability parameters for the developed HPLC-PDA method. Figure S1. UV–VIS spectra obtained by HPLC-PDA detection for (A) monacolin K and (B) monascorubrin. Figure S2. Representative peak purity plots of MK in RYR FSs: Sample 5 and Sample 12. Figure S3. HPLC–PDA chromatographic analysis of RYR FSs. Figure S4. GC–MS total ion chromatograms of selected RYR FSs samples # 5, 10, 12, 13 and 14 acquired in scan mode.

## Abbreviations

The following abbreviations are used in this manuscript:

σ	Standard deviation of the linear response
As	Symmetry factor
DUIS	Dual ion source
EFSA	European food safety authority
EMA	European medicines agency
EU	European union
FDA	Food and Drug Administration
FSs	Food supplements
GC-MS/MS	Gas chromatography tandem mass spectrometry
HPLC	High-performance liquid chromatography
HPLC-PDA	High-Performance Liquid Chromatography with Photodiode Array Detection
LC-MS	Liquid chromatography–mass spectrometry
LC-MS/MS	Liquid chromatography tandem mass spectrometry
LDL	Low-density lipoprotein
LDL-C	Low-density lipoprotein cholesterol
LOD	Limit of Detection
LOQ	Limit of Quantification
MRM	Multiple reaction monitoring
MK	Monacolin K
MS	Mass spectrometry
NDA	Panel on Dietetic Products, Nutrition and Allergies
NIST	National Institute of Standards and Technology
PDA	Photodiode array detector
QC	Quality control
RIs	Retention indices
RSD	Relative standard deviation
Rs	Resolution
RYR	Red yeast rice
S	Slope of the calibration curve
SIM	Selected ion monitoring
TIC	Total ion chromatogram
UHPLC-MS	Ultra-high-performance liquid chromatography–mass spectrometry
UV–VIS	Ultraviolet–visible spectroscopy
