# Supplementary material for: Quality and Authenticity Evaluation of Red Yeast Rice Food Supplements from the Bulgarian Market
Source: Foods. 2026 Oct 9;15(20):3583. doi: 10.3390/foods15203583 (PMC13702926; doi:10.3390/foods15203583)
Supplement: Supplementary file 1 [file foods-15-03583-s001.zip › foods-4585143-supplementary.pdf]

Supplementary Material

1 Supplementary Tables

Table S1. System suitability parameters for the developed HPLC-PDA method.

	Monacolin K	Monascorubrin
**Selectivity factor (α)**	–	2.15
**Resolution (Rs)**	–	19.63
**Number of theoretical plates (N)**	13915	16220
**Symmetry factor (As)**	1.05	0.98

2. Supplementary Figures

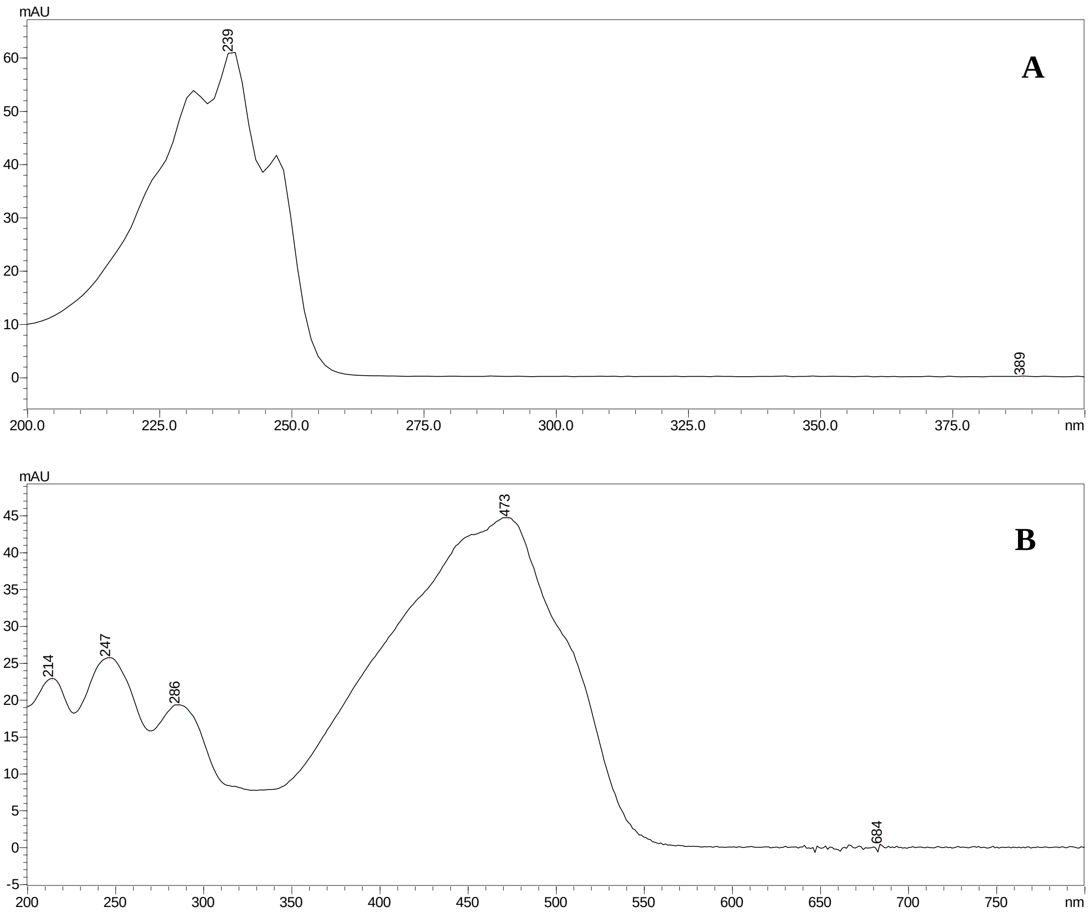

Figure S1. UV–VIS spectra obtained by HPLC-PDA detection for (A) monacolin K and (B) monascorubrin.

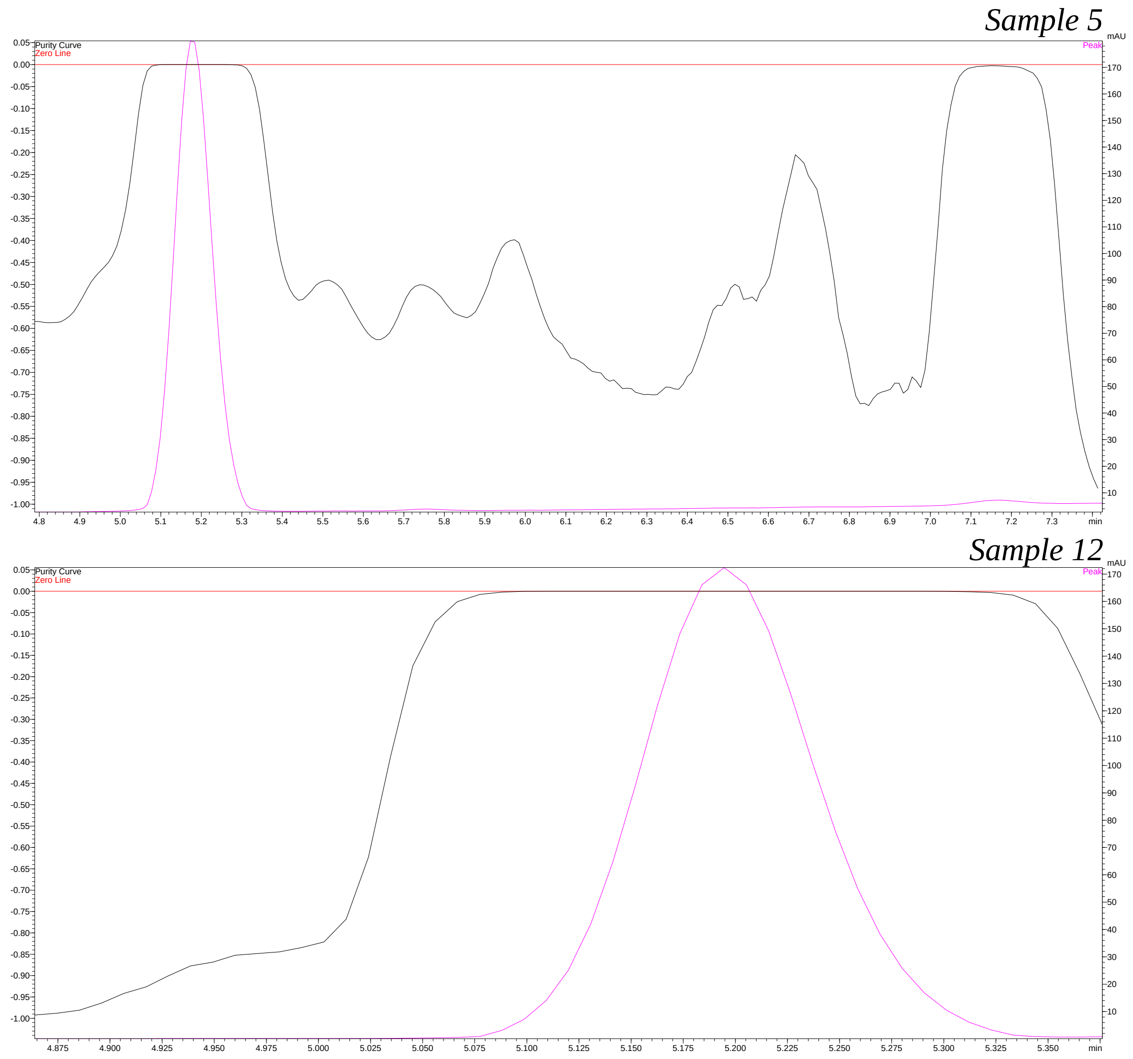

Figure S2. Representative peak purity plots of MK in RYR FSs: Sample 5 and Sample 12.

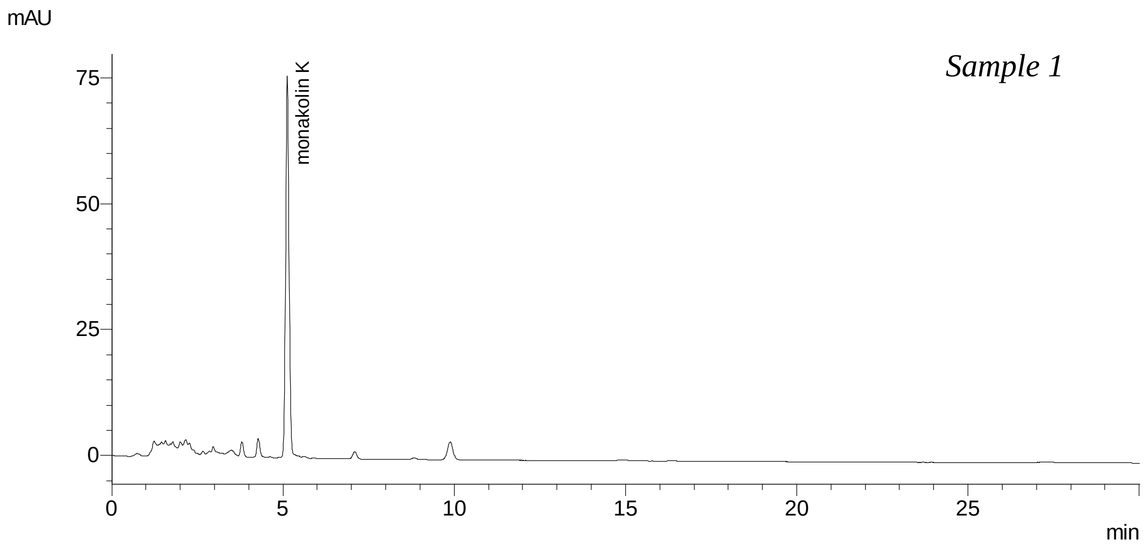

mAU
Sample 1
monakolin K
75
50
25
0
0
5
10
15
20
25
min

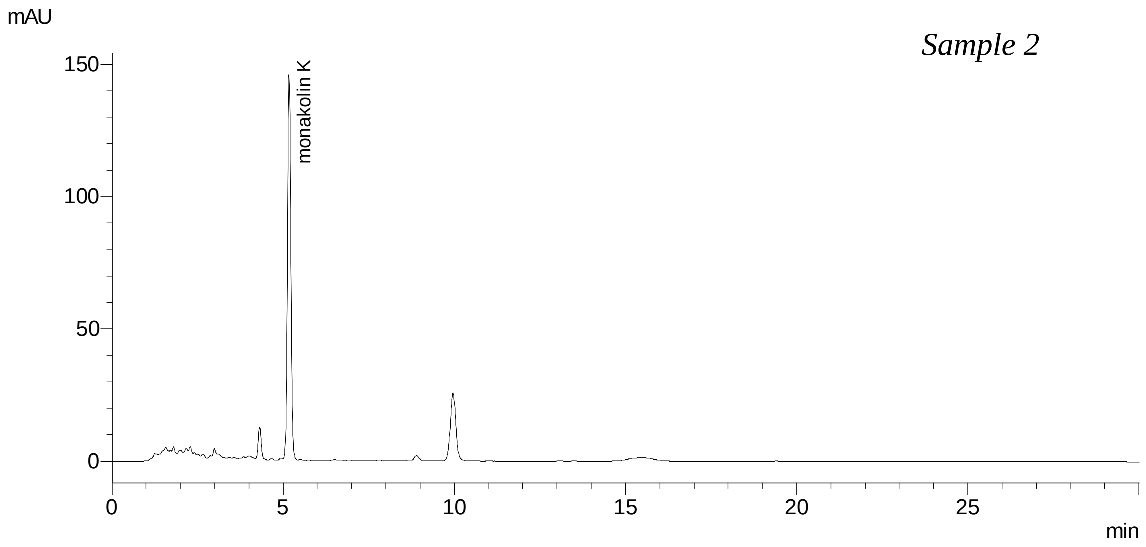

mAU
Sample 2
monakolin K
150
100
50
0
0
5
10
15
20
25
min

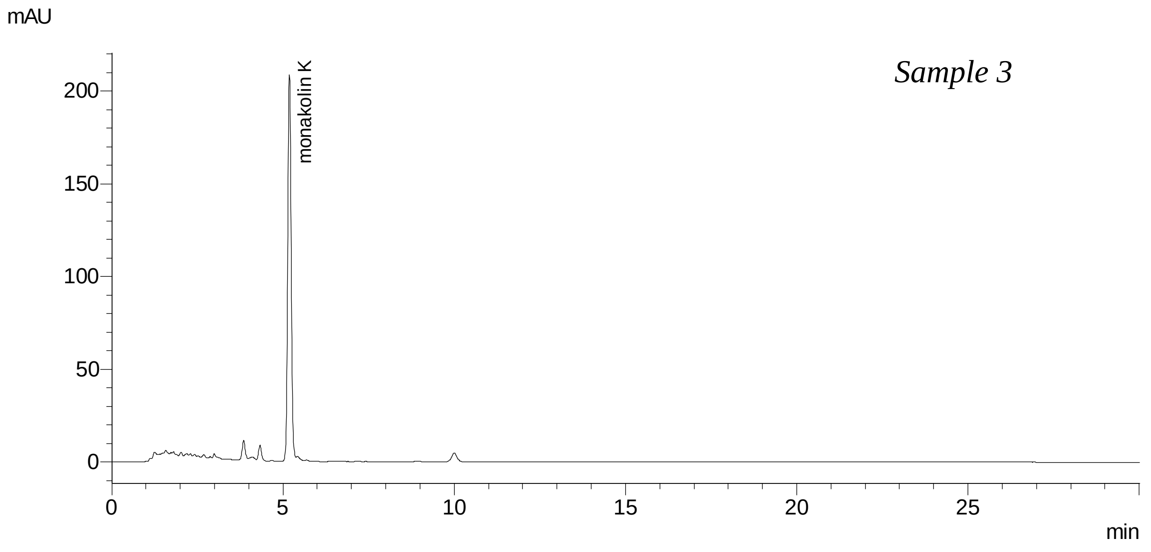
mAU
Sample 3
monakolin K
200
150
100
50
0
0
5
10
15
20
25
min

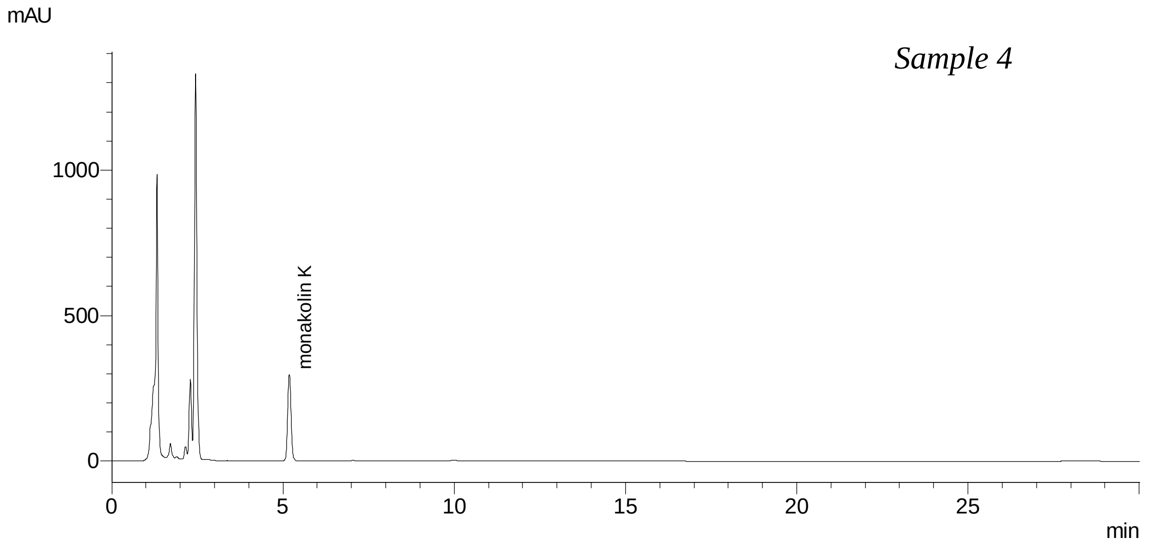
mAU
Sample 4
monakolin K
1000
500
0
0
5
10
15
20
25
min

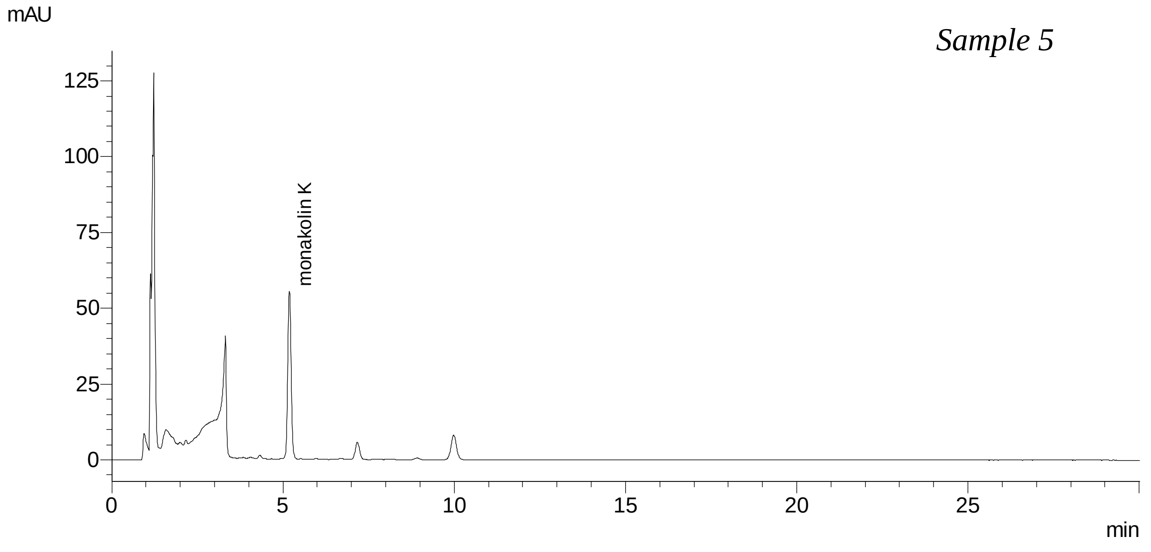
mAU
Sample 5
monakolin K
125
100
75
50
25
0
0
5
10
15
20
25
min

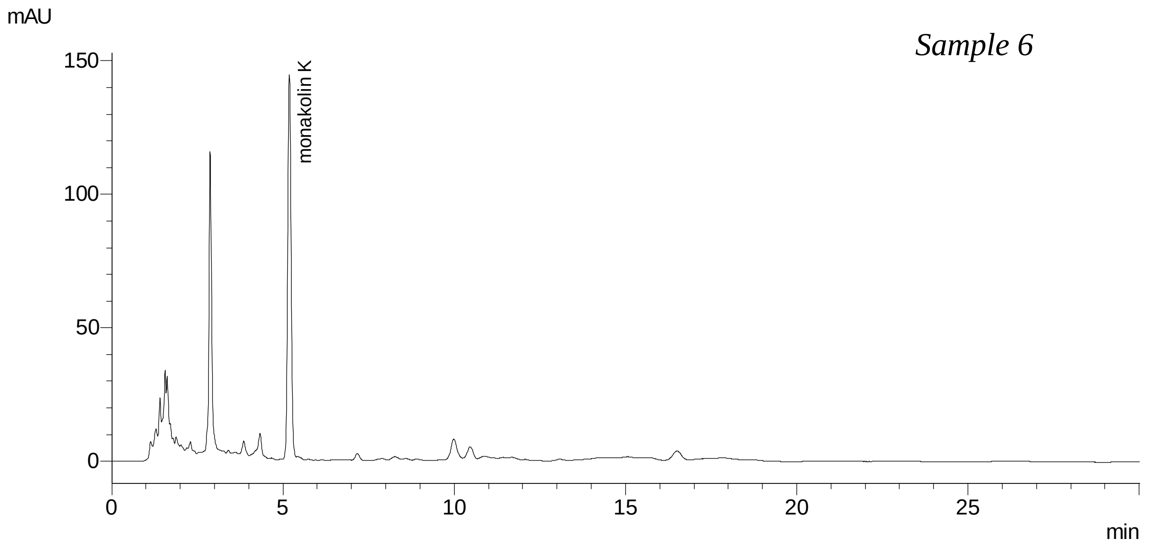
Sample 6
mAU
150
100
50
0
monakolin K
0
5
10
15
20
25
min

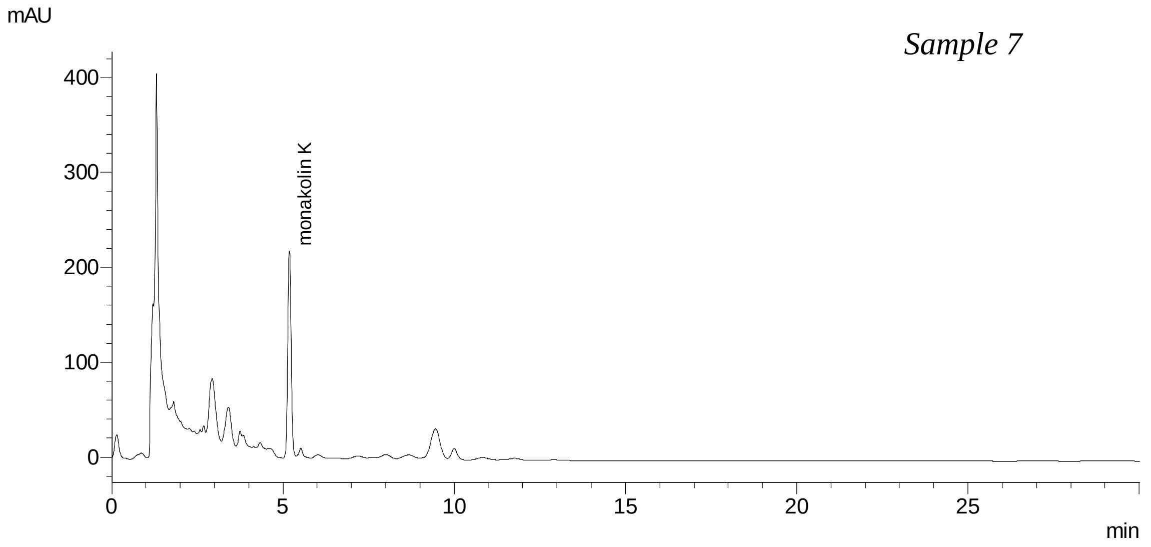
Sample 7
mAU
400
300
200
100
0
monakolin K
0
5
10
15
20
25
min

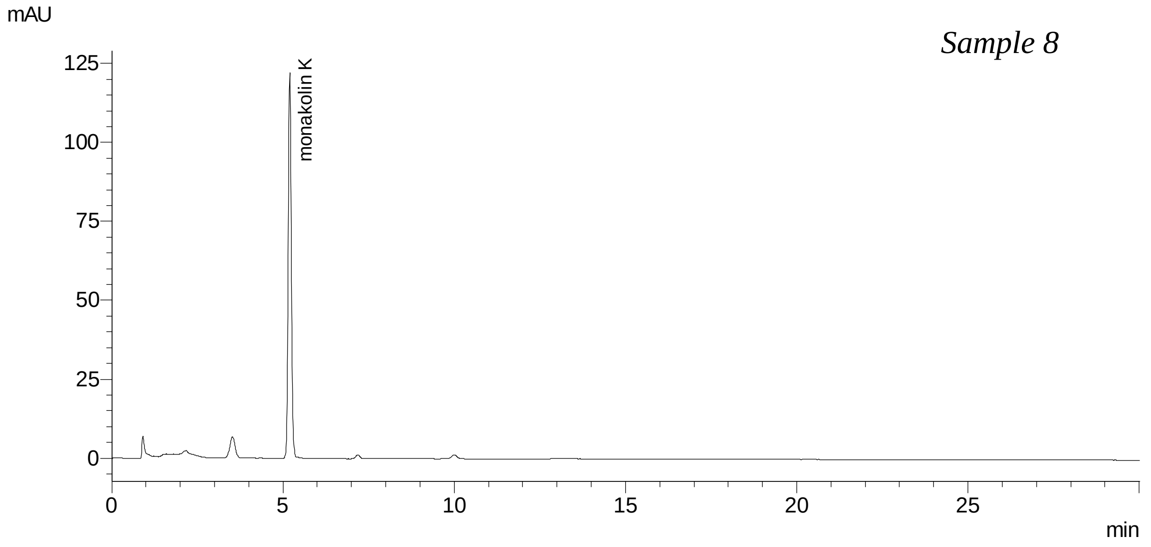
Sample 8
mAU
125
100
75
50
25
0
monakolin K
0
5
10
15
20
25
min

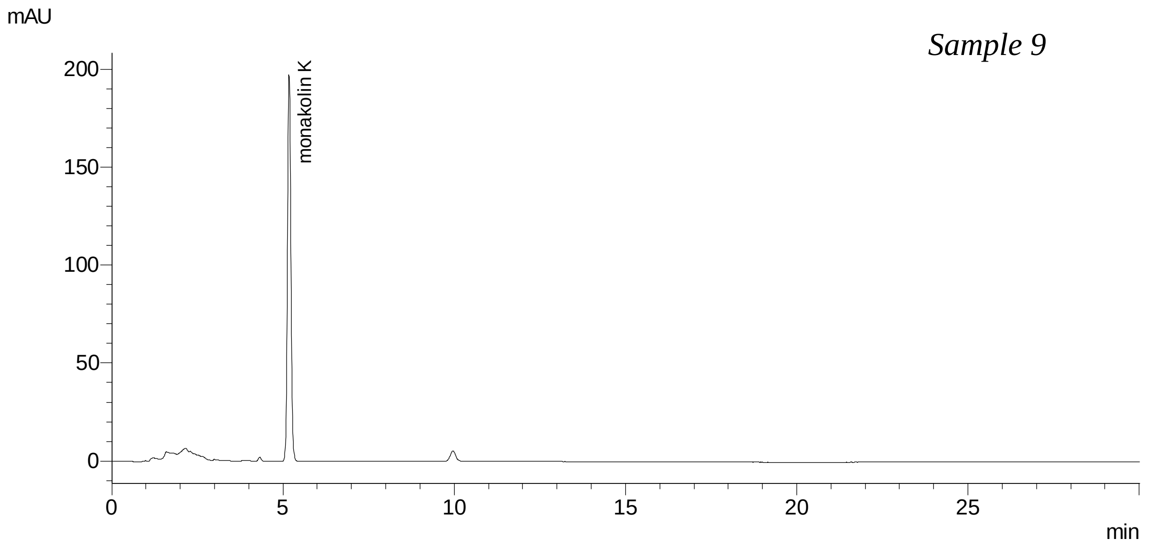
mAU
Sample 9
monakolin K
200
150
100
50
0
0
5
10
15
20
25
min

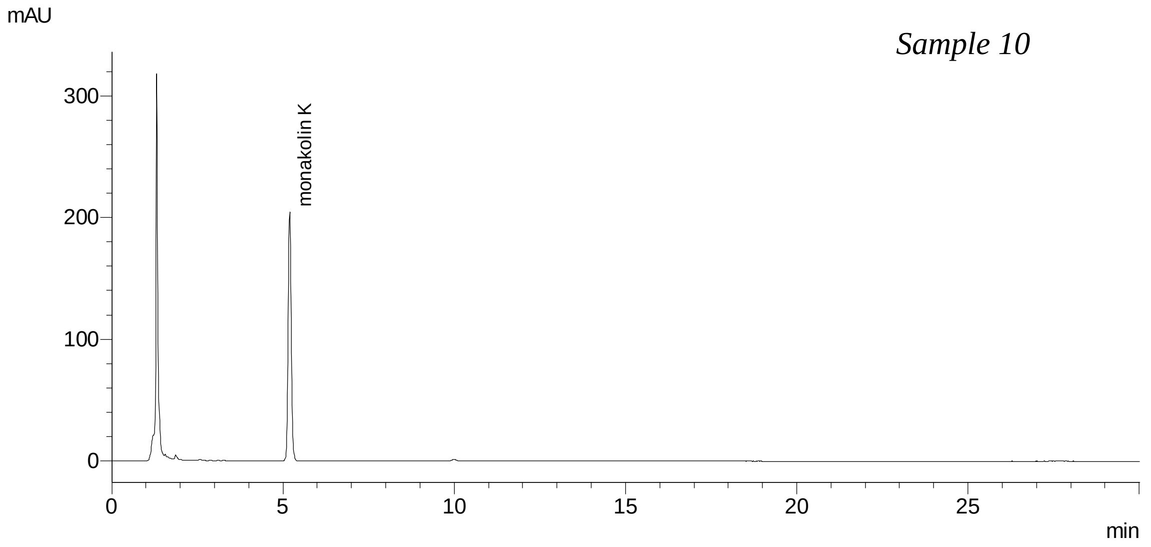
mAU
Sample 10
monakolin K
300
200
100
0
0
5
10
15
20
25
min

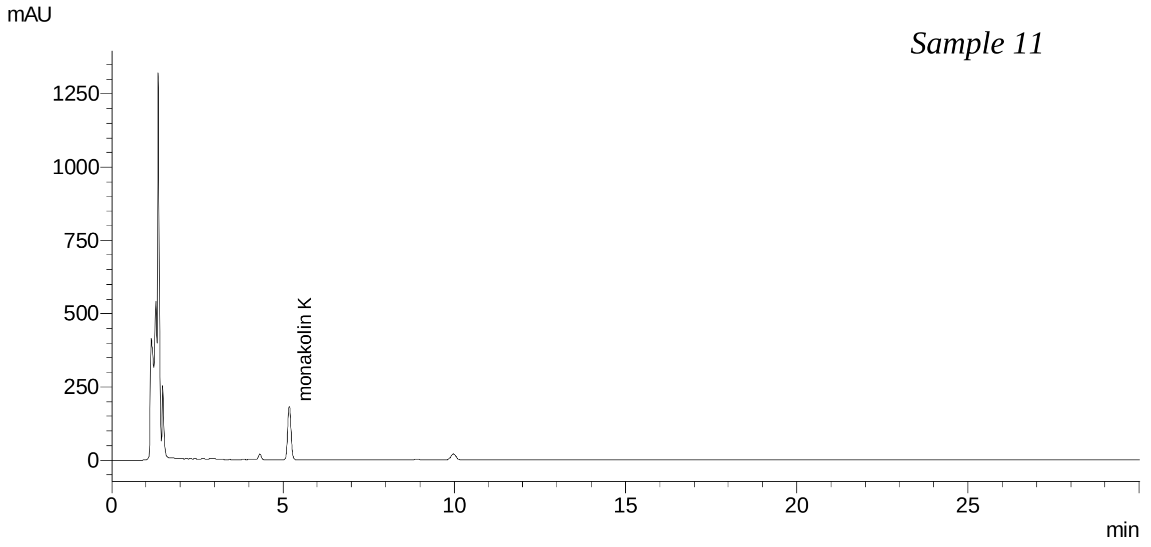
mAU
Sample 11
monakolin K
1250
1000
750
500
250
0
0
5
10
15
20
25
min

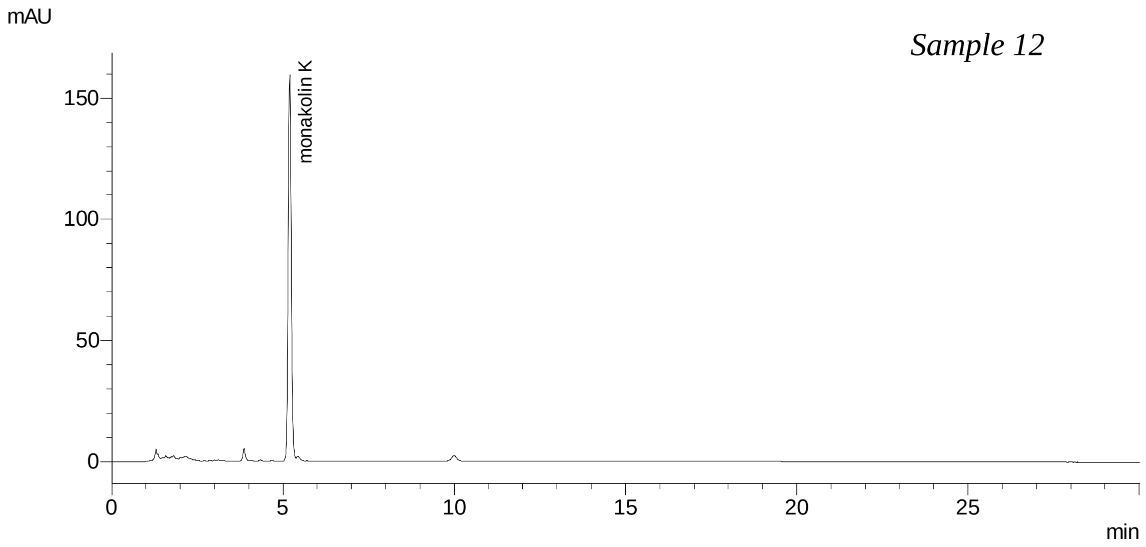
mAU
Sample 12
monakolin K
150
100
50
0
0
5
10
15
20
25
min

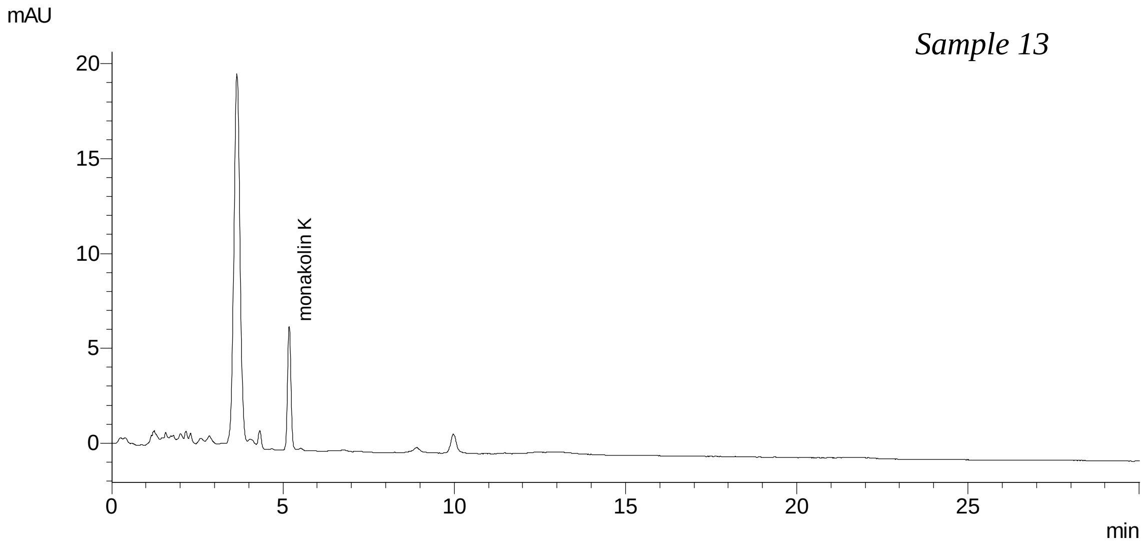
mAU
Sample 13
monakolin K
20
15
10
5
0
0
5
10
15
20
25
min

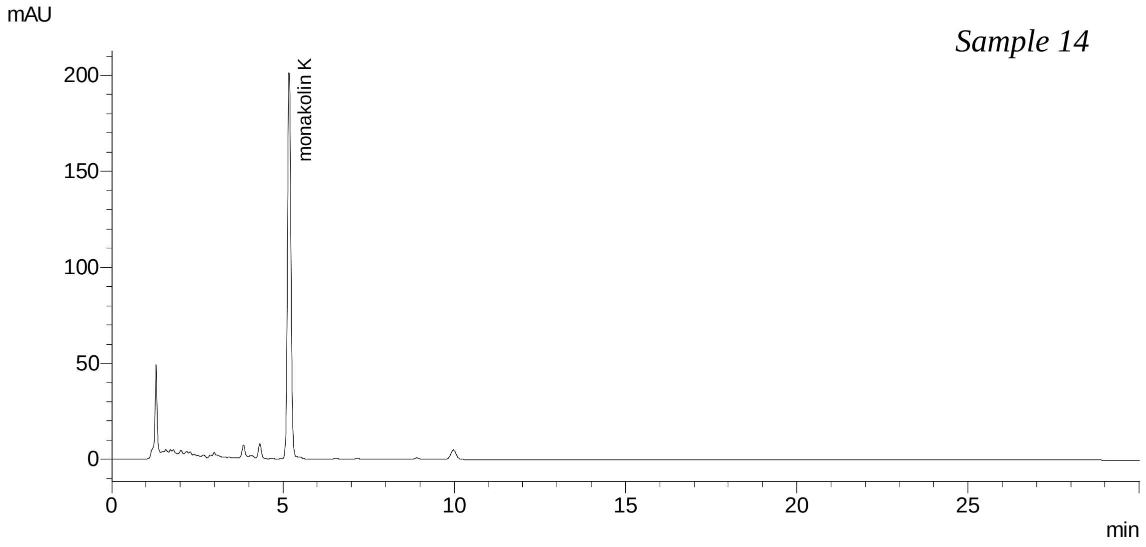
mAU
Sample 14
monakolin K
200
150
100
50
0
0
5
10
15
20
25
min

Figure S3. HPLC–PDA chromatographic analysis of RYR FSs.

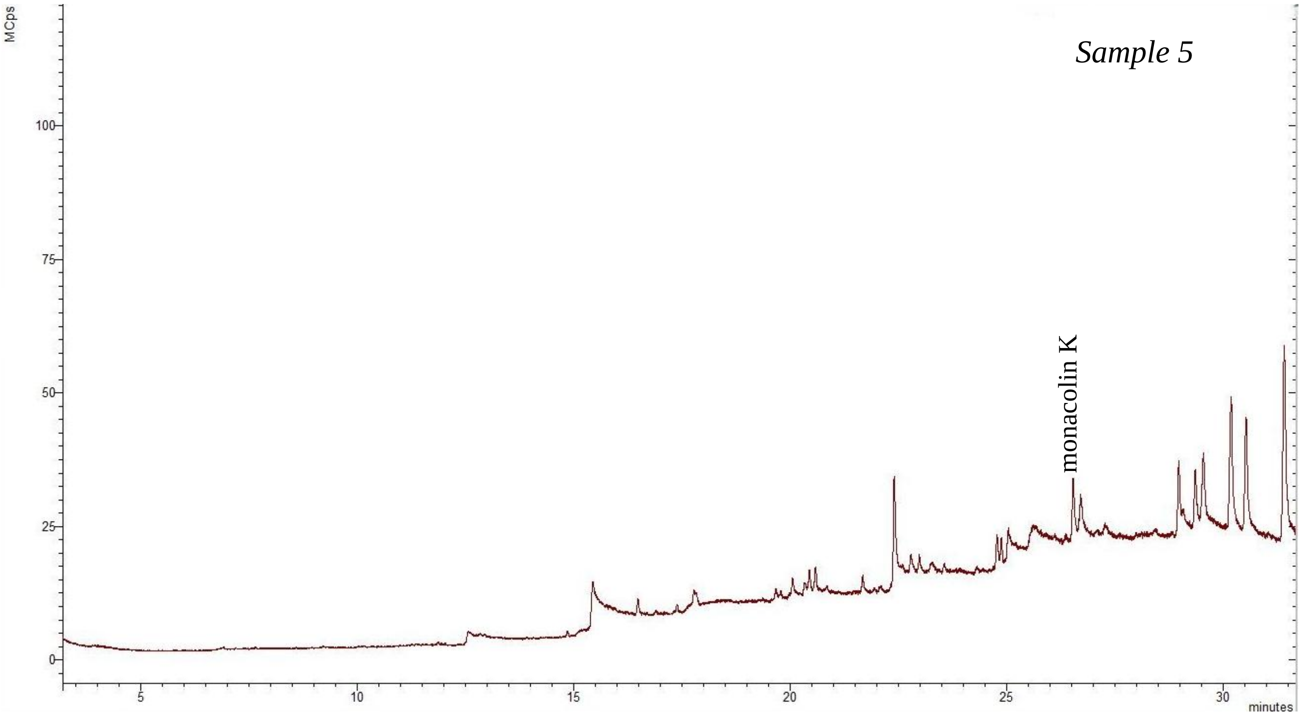

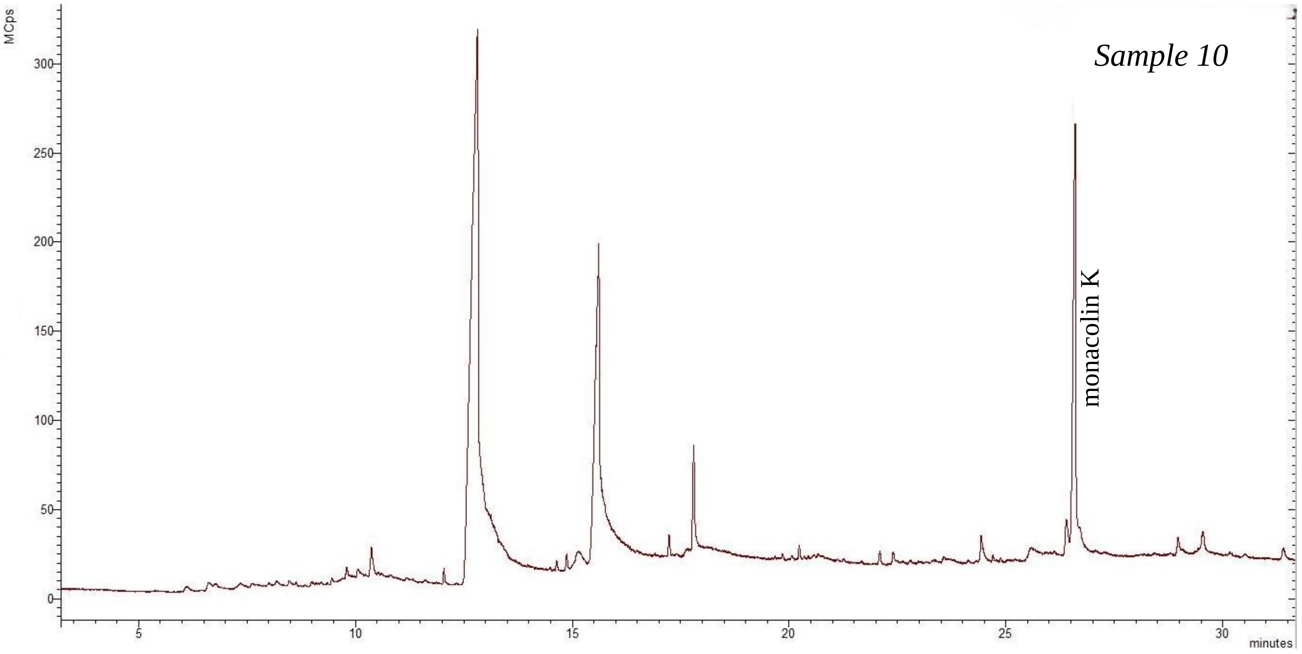

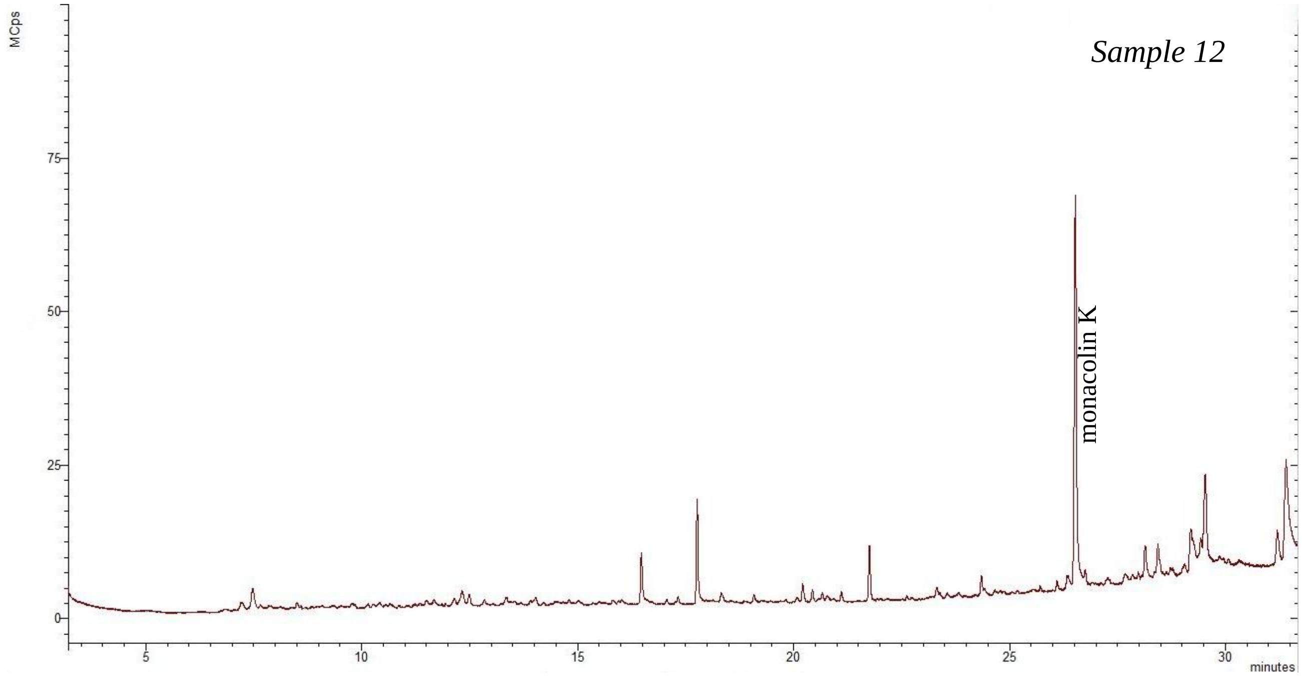
MCps
Sample 12
75
50
25
0
monacolin K
5
10
15
20
25
30
minutes

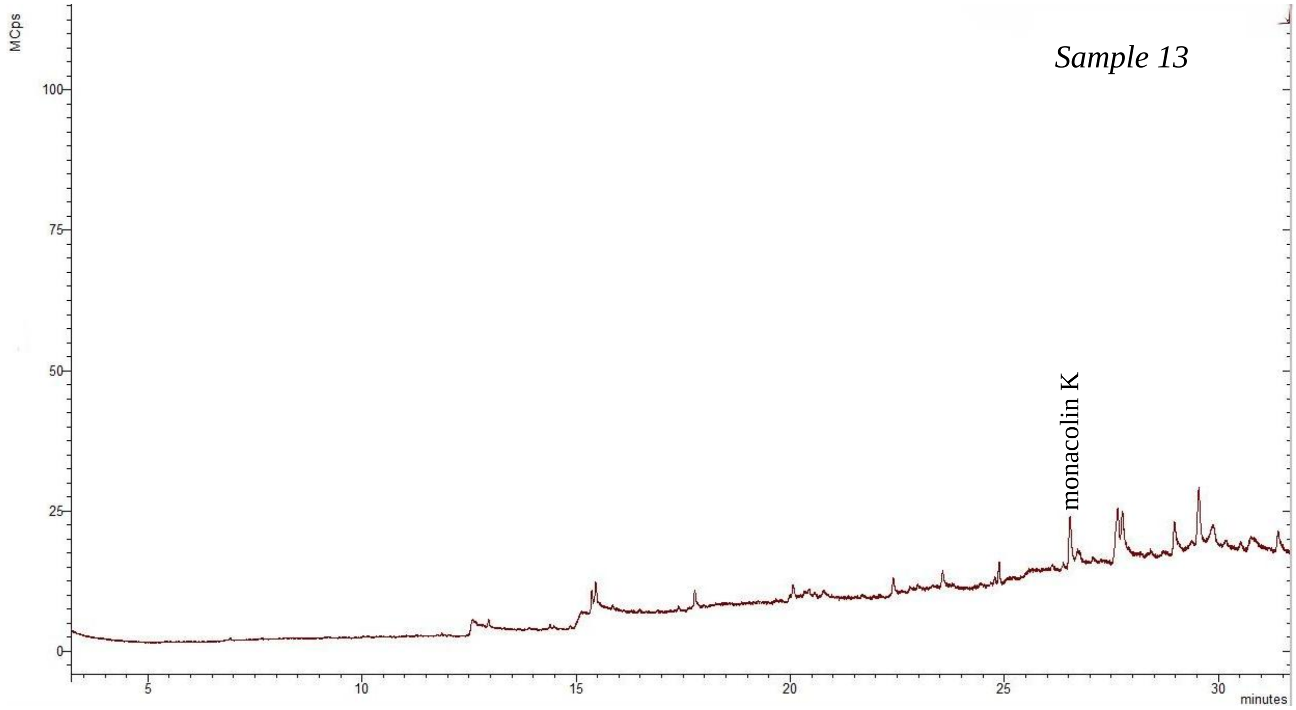
MCps
Sample 13
100
75
50
25
0
monacolin K
5
10
15
20
25
30
minutes

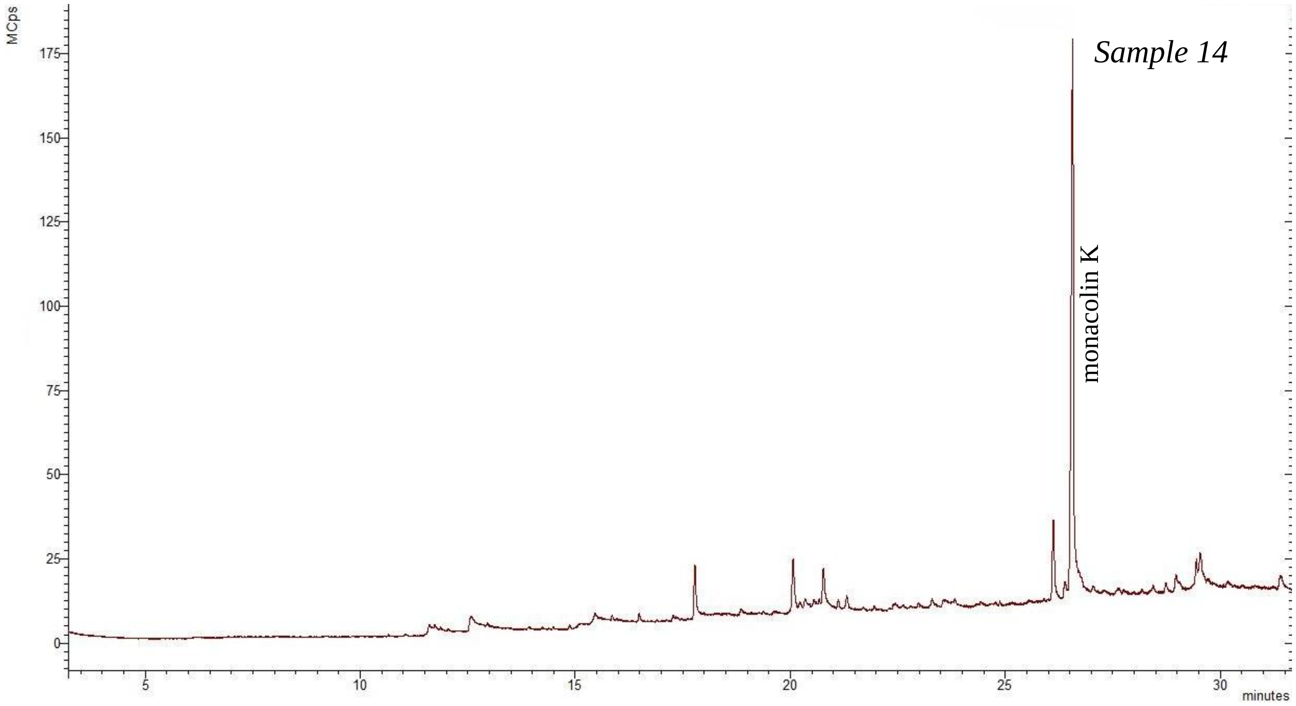

Figure S4. GC–MS total ion chromatograms of selected RYR FSs samples # 5, 10, 12, 13 and 14 acquired in scan mode.
